# Atypical Early Presentation of Multicentric Carpotarsal Osteolysis: Ankle Pain and Cavovarus Deformity Without Osteolysis

**DOI:** 10.7759/cureus.100954

**Published:** 2026-01-06

**Authors:** Daniel Kim, Marty McGraw, Jennifer Kucera, Tushar Chandra, Manish Bajaj

**Affiliations:** 1 Radiology, Nemours Children's Hospital, Orlando, USA; 2 Pediatric Radiology, Nemours Children's Hospital, Orlando, USA; 3 Neuroradiology, Nemours Children's Hospital, Orlando, USA

**Keywords:** idiopathic multicentric osteolysis, juvenile idiopathic arthritis (jia), multicentric carpo-tarsal osteolysis, multicentric carpotarsal osteolysis (mcto), multicentric osteolysis

## Abstract

Multicentric carpotarsal osteolysis (MCTO) is a rare skeletal dysplasia classically associated with carpal and tarsal osteolysis. Its presentation is highly variable, ranging from subtle musculoskeletal deformities to progressive bone loss and renal involvement. We report a nine-year-old female who presented with progressive ankle pain, cavovarus foot deformity, and gait disturbance. Initial radiographs demonstrated osteopenia and bilateral pes cavus without frank osteolysis. Genetic testing identified a pathogenic MAFB variant, confirming the diagnosis of MCTO.

This case highlights the broad clinical and radiologic spectrum of MCTO, in which early or atypical presentations may have subtle, nondiagnostic imaging findings. Recognizing these early features can raise clinical suspicion and prompt genetic evaluation, helping to avoid misdiagnosis and unnecessary interventions. Awareness of the broad spectrum of clinical and radiographic findings is essential for timely diagnosis and appropriate management.

## Introduction

Multicentric carpotarsal osteolysis (MCTO) is a rare hereditary skeletal disorder characterized by progressive osteolysis of the carpal and tarsal bones, often associated with nephropathy, with fewer than 60 documented cases as of 2022 [1]. Most cases of MCTO present in early childhood with advanced bone resorption, manifested as joint pain, swelling, deformity, and radiographic signs of aggressive osteolysis of the carpal and tarsal bones. In contrast, early or subtle presentations pose a diagnostic challenge, as imaging findings can mimic benign orthopedic variations, metabolic bone disorders, or inflammatory arthropathies [2]. The clinical and radiographic spectrum is broad. Some patients initially exhibit nonspecific findings, such as osteopenia, deformity, or gait abnormalities, before obvious osteolysis is seen [3]. A literature review of 54 reported cases revealed a mean age of symptom onset of two years and a mean age at diagnosis of nine years, demonstrating an average delay of seven years [1]. Recognizing these subtler manifestations is important, as delayed diagnosis can lead to inappropriate treatment and missed opportunities for early surveillance of systemic complications, particularly renal function. 

We present the case of a nine-year-old with progressive foot deformities that highlights the early spectrum of symptoms in MCTO and the importance of considering MCTO in the differential diagnosis of pediatric patients presenting with nonspecific joint pain, in the absence of radiographic osteolysis.

## Case presentation

**Figure 1:**
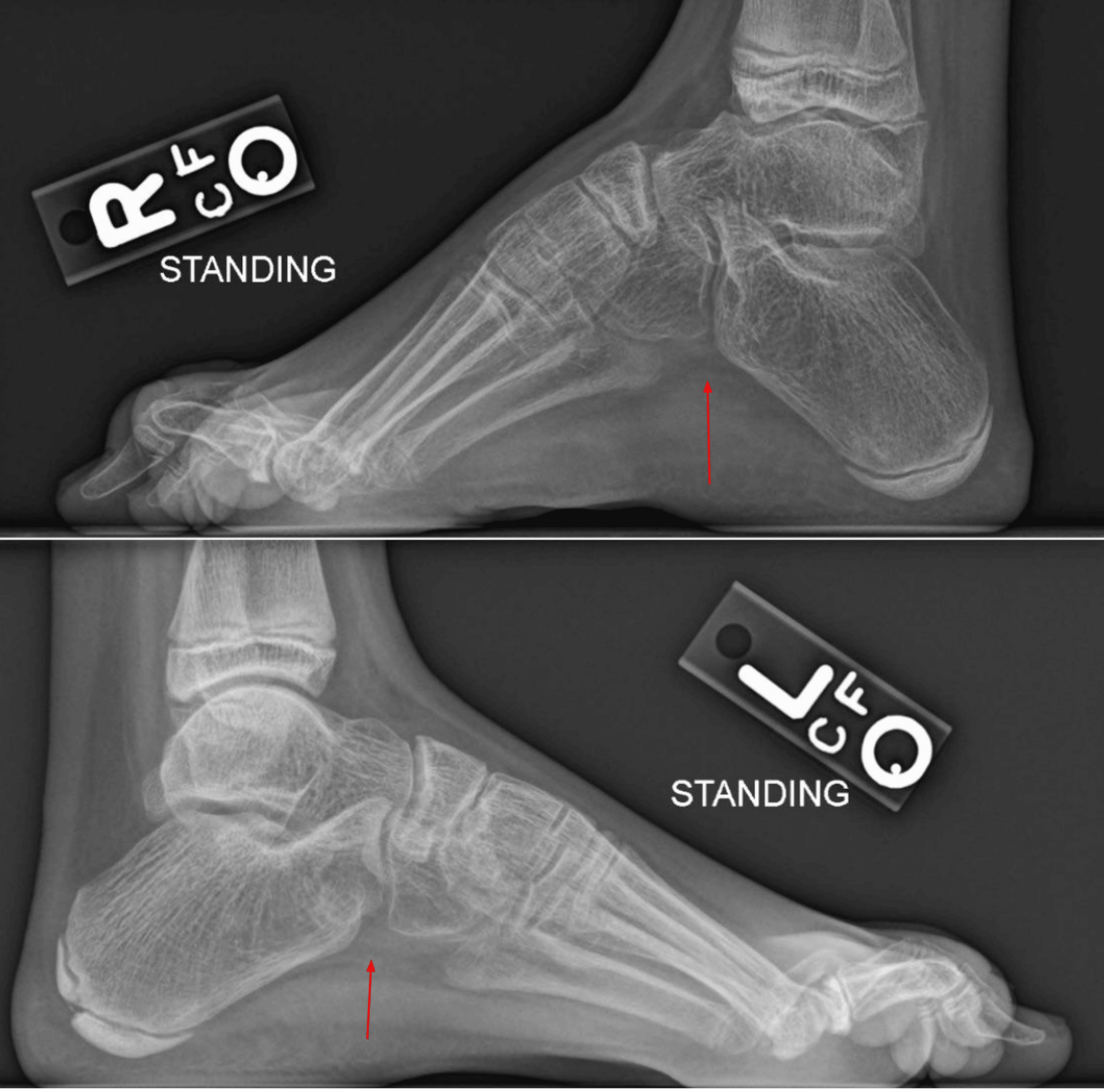
Initial radiographs of bilateral feet show pes cavus, right worse than left, and generalized osteopenia. Red arrows depict bilateral pes cavus.

A nine-year-old female with no significant past medical history presented to neurology for progressive bilateral foot deformities, neuropathic pain, and gait abnormalities that began around four years of age. The initial radiograph was nonspecific and revealed generalized osteopenia with bilateral pes cavus, right worse than left (Figure 1). Further workup included electromyography, magnetic resonance imaging of the spine, a comprehensive neuropathy genetic panel, and serum inflammatory markers, all of which were normal.

**Figure 2:**
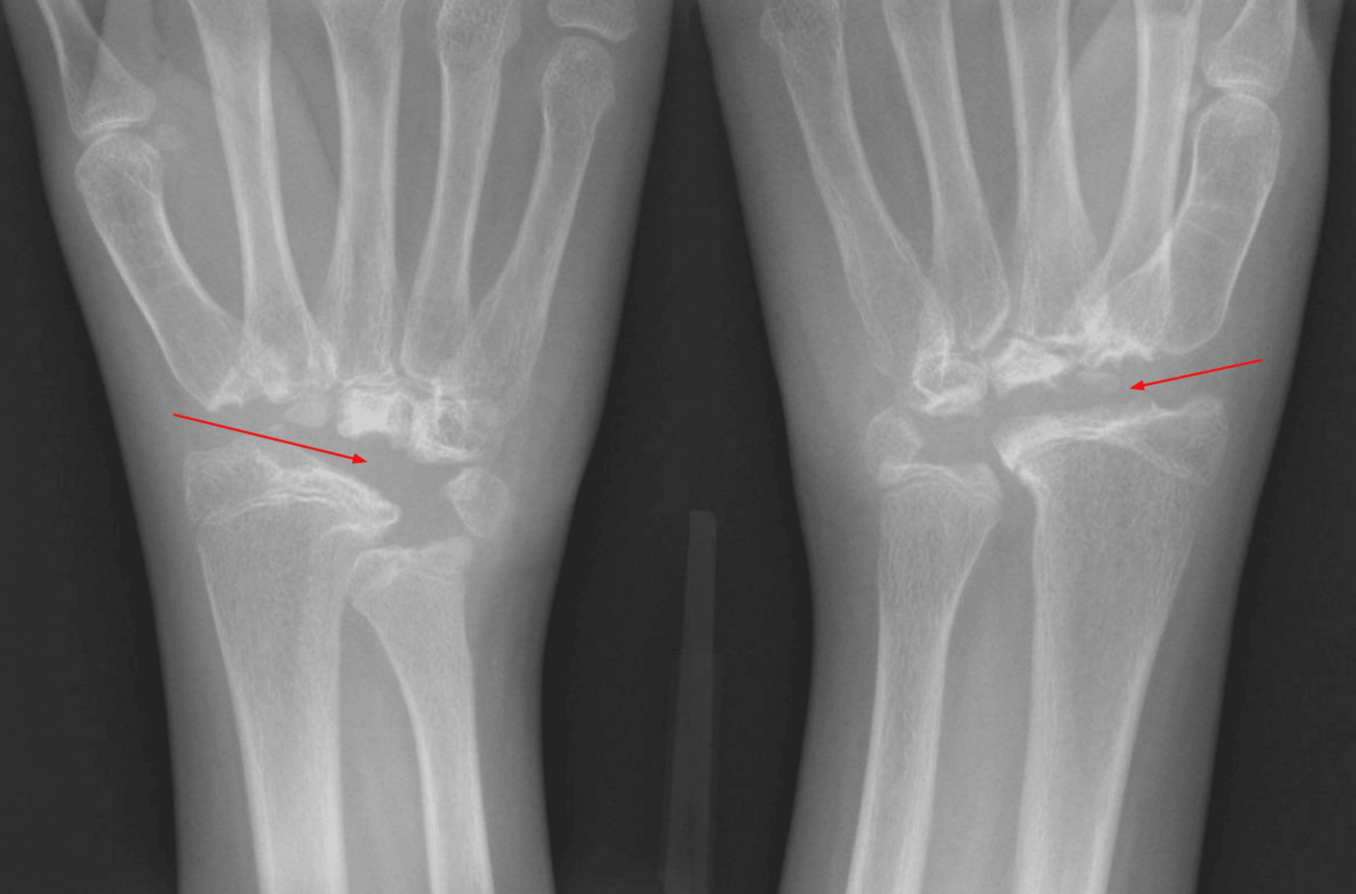
Radiographs of the bilateral wrists show extensive osteolysis of the carpal bones, with degenerative changes at the carpometacarpal joints. Red arrows depict bilateral osteolysis of carpal bones.

The patient was then referred to orthopedic surgery to address the pain and functional impairment associated with the right cavovarus deformity. At the same time, whole-exome genetic testing was ordered and revealed a heterozygous pathogenic variant in the MAFB gene, confirming the diagnosis of MCTO. The patient underwent surgical reconstruction of the right foot and aggressive physical therapy. She eventually reported improved function and minimal foot pain without the use of orthotics. At age 11, the patient presented again with bilateral wrist pain. She slowly began to lose function and mobility in her wrists and had difficulty closing her fists, playing the violin, and combing her hair. Radiographs of the bilateral wrists demonstrated diffuse osteolysis of multiple carpal bones, with degenerative changes at the carpometacarpal joints (Figure 2). No definitive treatment was offered for her wrists due to her known diagnosis of MCTO. Fusion of the wrists is being considered once she is skeletally mature. She currently does not have any renal complications, but will continue to be monitored annually. 

## Discussion

MCTO is a rare skeletal dysplasia, most commonly caused by heterozygous mutations in the MAFB gene. Pathogenic variants disrupt transcriptional regulation of osteoclast differentiation, leading to progressive bone resorption. MCTO can also impact renal podocytes, resulting in proteinuria or nephrotic syndrome in some patients [4]. It remains diagnostically challenging due to its rarity and variable presentation. The age of onset typically falls in early childhood, but symptoms can range from subtle joint discomfort to severe pain and deformity. Some patients present with progressive swelling, pain, and stiffness, while others may remain relatively asymptomatic until functional impairment becomes evident, at which point significant osteolysis is already apparent on imaging [5].

Early radiologic findings, as seen in our case, may present as generalized osteopenia, subtle irregularity, or mild resorption/collapse of the carpal and tarsal bones. These nonspecific findings are often bilateral and symmetrical, and mimic inflammatory changes seen in juvenile idiopathic arthritis (JIA) [3]. Other cases of MCTO, with nonspecific polyarticular pain without definitive carpal or tarsal bone resorption, are often misdiagnosed as JIA, contributing to diagnostic delay. Outside of joint pain, MCTO can further be distinguished by renal, ocular, or craniofacial abnormalities [3]. As MCTO progresses, findings of advanced disease are overt, and show complete disappearance of the carpal and tarsal bones, leading to joint instability, deformity, and loss of function [6]. Just like in our case, a few years after the initial diagnosis, the patient developed near-complete osteolysis of both wrists. Nevertheless, imaging alone is insufficient in early disease, and genetic confirmation is necessary. 

Treatment for MCTO is primarily supportive, as many patients present with advanced disease. However, denosumab has been proposed, in case reports, as a potential therapy aimed at preventing osteolysis [7]. Other reports state that denosumab does not stop the progression of disease [8]. Specific factors regarding the decision to start the medicine were not available for review, and there is not enough evidence of standardized treatment guidelines. In our case, the patient underwent surgical reconstruction for her right cavovarus deformity before being diagnosed with MCTO. At the time, the absence of frank osteolysis and the presence of foot deformity supported a more common orthopedic etiology. She reported subjective improvement of her symptoms and gait following repair; however, in retrospect, this highlights a potential risk of surgical management in patients with underlying osteolytic disorders, as there may be further complications and altered biomechanics in relation to progressive tarsal osteolysis, with retained metallic hardware in the foot.

## Conclusions

In summary, MCTO is a rare but important consideration in pediatric patients with progressive musculoskeletal symptoms and nonspecific radiographic findings. Our case aims to highlight the atypical, early presentation of MCTO and emphasize that the absence of overt osteolysis does not exclude the diagnosis. In these situations, a comprehensive approach that integrates clinical suspicion, careful radiologic evaluation, and genetic testing ensures an accurate diagnosis of MCTO and facilitates early monitoring for systemic complications.
